# Effects of Rheumatoid Arthritis on the Larynx

**DOI:** 10.22038/ijorl.2020.43213.2418

**Published:** 2020-05

**Authors:** Mehdi Dehghan, Akram Ahmadi, Behnaz Yousefghahari, Keyvan Kiakojouri, Hemmat Gholinia

**Affiliations:** 1 *Department of Speech Therapy, School of Rehabilitation, Babol University of Medical Sciences, Babol, IR, Iran.*; 2 *Clinical Research Development Unite of Rouhani Hospital, Babol University of Medical Sciences, Babol, IR, Iran.*

**Keywords:** Larynx, Rheumatoid arthritis, Voice, Vocal cords

## Abstract

**Introduction:**

The aim of the present study was to compare the videolaryngostroboscopic findings between patients with rheumatoid arthritis and vocally healthy controls.

**Materials and Methods:**

This case-control descriptive study was performed on 113 people, including 50 patients with rheumatoid arthritis and 63 controls. The participants were subjected to videolaryngostroboscopic examinations in order to evaluate fundamental frequency, different structural vocal lesions, patterns of glottal closure, subglottal changes, supraglottis appearance, and movement patterns of the arytenoid cartilage. The obtained results were compared between the two research groups. Data analysis was performed in the Statistical Package for the Social Sciences, version 24.0. A p-value less than 0.05 was considered statistically significant.

**Results:**

The results revealed a statistically significant difference between the two groups in terms of the complete pattern (P=0.00) and strained state of glottal closure (P=0.00), pattern of subglottal changes (χ^2^=25.98, df=2; P<0.001), and movement patterns of the arytenoid (χ^2^=21.16, df=1; P<0.001). Additionally, based on the obtained frequencies, the two groups showed significant differences regarding the normal state of the larynx (P=0.00), hypertrophy of vocal fold (P=0.007), epithelial change (P=0.007), and Reinke's edema (P=0.001). However, the videolaryngostroboscopic examination results revealed no significant difference between the two groups in terms of polyp (P=0.20), nodule (P=0.57), sulcus vocalis (P=0.08), cyst (P=0.45), and atrophy of vocal folds (P=0.45).

**Conclusion:**

It seems that rheumatoid arthritis affects the patterns of arytenoids movement, some kinds of glottal closure patterns, and subglottal changes. As the results indicated, the occurrence of some laryngeal structural changes was higher in patients with rheumatoid arthritis than in individuals without this disorder.

## Introduction

Rheumatoid arthritis (RA) is considered an autoimmune inflammatory chronic disease that causes serious damages in the joints and destruction in the bones. The etiology of RA is unknown, and its prevalence is estimated at 1% (1). The inflammatory changes in this disease happen progressively and persistently and affect different body organs adversely (2). The larynx as an organ that is responsible for phonation mechanism is also adversely influenced by this disease (3).

Some epidemiological studies have reported a higher prevalence of dysphonia in patients with RA (3,4). In a cohort study, Speyer et al. investigated the prevalence of dysphonia in patients with RA in comparison with that in vocally healthy people. In doing so, they asked the patients to complete the health-related quality of life questionnaire. They reported a prevalence rate of 12-27% for dysphonia in patients with RA; however, this rate was estimated at 3-8% in the control group (3). Fisher et al. studied voice disorders in patients with RA. To do so, they asked the RA patients and control group to complete the Voice Handicap Index-10 (VHI-10). Their findings indicated a higher VHI-10 score for patients with RA than for the control group (4). Roy et al. also conducted a study to investigate the prevalence of voice disorders in patients with RA. In the mentioned study, the participants filled out the short form-36 and Voice-Related Quality of Life questionnaires. They reported a dysphonia prevalence of 35% in patients with RA (5). It seems that several factors may contribute to dysphonia in individuals who suffer from RA (3). Involvement of the cricothyroid and cricoarytenoid joints is the main cause of the emergence of vocal symptoms in this population (6,7). A number of studies have documented the existence of some vocal cord lesions like bamboo nodes and vocal fold rheumatoid nodules in patients with RA (3,8-10). The adverse effects of inflammatory changes are also observed in the intrinsic muscles of the larynx (3). It seems that the above-mentioned changes lead to dysphonia in patients with RA (2). Several procedures have been used for the evaluation of voice manifestations and structural changes of the larynx in this population. Some of the procedures include acoustic analysis (7), high-resolution computerized tomography (7), histopathological investigation (6), and examination of the larynx by videolaryngo- stroboscopy (7,11,12). 

Videolaryngostroboscopic examination as the visual procedure to study the larynx has some advantages. This approach is user-friendly and provides vivid and sharp images from the larynx (13). Several studies have been carried out to analyze the videolaryngostroboscopic findings in patients with RA. Beirith et al. studied the laryngeal changes in 48 patients with RA by videolaryngoscopy procedure. They observed no statistically significant difference between the findings of the patients with RA and control group in terms of some structural changes like vocal fold nodules, unilateral vocal fold paralysis, and presbylaryngitis. However, there was a significant difference between the findings of the two groups regarding the glottic cleft and posterior laryngitis (11). 

In another study, Berjawi et al. used a laryngeal endoscopic examination to evaluate the patients with RA. They reported that this method could facilitate the observation of decreased mobility in vocal folds in only 10% of the patients with RA. Nonetheless, they reported moderate to severe edema in 27.3% of the patients with RA and 0% of the control group. No statistically significant difference was reported between the RA and control groups in terms of the edema of the vocal folds (7). Castro et al. investigated the manifestation of voice disorder by videolaryngostroboscopy in patients with RA (14). This procedure revealed the overriding of arytenoids in 7 out of 27 patients with RA which was the only statistically different variable in patients with RA, compared to that in the control group.To the extent of the researchers’ knowledge, no study has addressed the effects of RA on glottal closure patterns, arytenoids movement patterns, subglottal changes, supraglottis appearance, and different types of structural changes yet. Moreover, it is not still clear if the frequency of laryngeal structural changes in patients with RA is higher than that in the general population. With this background in mind, the present study was conducted to compare the fundamental frequency, pattern of glottal closure, subglottal changes, and different types of structural changes. This study also addressed the arytenoid movement patterns. 

## Materials and Methods

**Study design**

A case-control and descriptive study was performed to compare the videolaryngo- stroboscopic findings between patients with RA and healthy controls.

***Ethical issues***

The current study received an ethics code from the Babol University of Medical Sciences, Babol, Iran (Ref. code: MUBABOL. REC. 1395.29). All of the participants signed and completed the consent form to participate in this project before enrolling in the study.

Participants

The study population corresponded to a group of 50 patients with RA having a history of 5 years of illness and a control group consisting of 63 people with no sign of RA that were matched in terms of age. The research participants were selected using the convenience sampling technique. The patients were recruited from the Rheumatology Clinic of Rouhani Hospital of Babol in 2017-2018. The diagnosis of RA was based on the 2010 rheumatoid arthritis classification criteria that were suggested by the American College of Rheumatology/European League Against Rheumatism Collaborative Initiative (15). 

The control group was comprised of the staff of Babol University of Medical Sciences. The exclusion criteria for both groups included a history of laryngeal surgery, thyroidectomy, neurologic diseases, acute infections, tracheostomy, or traumatic brain injury.

***Research Procedure***

The videolaryngostroboscopic evaluation of the participants was carried out by digital videolaryngostroboscopy (set Endo-Digi Strobo-LED ECLERIS) using the PROCAM FULL PAL with a high-resolution module and high-speed digital imaging (HSDI) technique, allowing to register an image of vocal fold vibrations. 

The clear and vivid videos taken from each participant were analyzed frame by frame using the digital data capturing system by two experienced otorhinolaryngologists to investigate the movement patterns of the thyroarytenoid cartilages, type of glottal closure, patterns of subglottal changes, and structural changes in the larynx.

The collected demographic information included the age and gender of the participants. Videolaryngostroboscopic findings of the larynx were categorized into normal, nodule, polyp, Reinke's edema, hypertrophy of vocal folds, epithelial changes, atrophy of vocal folds, sulcus vocalis, and cyst. For the symmetry in arytenoid movement, we defined two variables, including overriding and symmetric movement. In addition, five states were considered for the glottal closure, including complete closure, posterior chink, oval shape, hourglass shape, and strained state. Fundamental frequency was also calculated and compared between the two groups. To calculate the fundamental frequency, the participants were asked to produce sustained */a/* with the habitual loudness and pitch.

Statistical analysis

Statistical Package for the Social Sciences, version 24.0 (SPSS, Inc., Chicago, IL) was used for the statistical analysis of this study. To compare the videolaryngostroboscopic findings, including arytenoid movement patterns, type of glottal closure, and subglottal changes, between the two groups, the Chi-square test was run. Furthermore, for some variables that the expected frequency was 0 or more than 20% of cells had expected frequencies fewer than 5, Fisher’s exact test was utilized. Independent sample t-test was also used to compare fundamental frequency between the RA and vocally healthy groups. The qualitative variables were presented as frequency and percentage, and fundamental frequency was ***described*** ***as*** mean and standard deviation. A p-value less than 0.05 was considered statistically significant (P<0.05). 

## Results

**Participants**

Demographic information of the participants is presented in Table 1. The present study included 50 patients with RA and 63 controls. Out of 50 patients in the RA group and 63 cases in the control group, 42 (37.2%) and 40 (35.4%) subjects were female, respectively. Accordingly, the two groups were comparable in terms of age (P=0.68). 

**Table 1:** Demographic characteristics of the participants

**Demographic characteristics**	**Patients with rheumatoid arthritis**	**Control group**
	n (%)	n (%)
Number of participants in each group	50 (44)	63 (56)
Gender	Female: 42 (37.2) Male: 8 (7.1)	Female: 40 (35.4) Male: 23 (20.4)
Age (year) Mean±standard deviation	53.42±11.02	53.74±10.89

Videolaryngostroboscopic findings of rheumatoid arthritis and control groups

Videolaryngostroboscopic findings related to the structural changes of the participants are provided in Table 2 as given by the frequencies cross-tabulated in Table 2, the frequencies of the nodule diagnosis were not significantly different (P=0.57). In terms of pathology, the frequencies of polyp diagnosis were not significantly different between the two groups (P=0.20). Furthermore, there was no statistically significant difference between the two groups regarding the cyst (P=0.45), sulcus vocalis (P=0.08), and atrophy of the vocal folds (P=0.45) findings. However, the frequencies of normal structure in the larynx showed that the two groups were statistically different (P=0.00). In addition, the two groups were significantly different regarding the frequency of the diagnosis of Reinke's edema (P=0.00) and hypertrophy of vocal folds (P=0.007), as well as epithelial changes (P=0.007).

**Table 2:** Videolaryngostroboscopic findings of patients with rheumatoid arthritis and control group

**Videolaryngostroboscopic findings**	**Patients with rheumatoid arthritis**	**Control group**	**P-value**
	**Frequency (%)**	**Frequency (%)**	
Normal	17 (34)	60 (95.2)	0.00
Nodule	3 (6)	3 (4.8)	0.57
Polyp	2 (4)	0 (0)	0.20
Reinke's edema	11 (22)	0 (0)	0.001
Hypertrophy of vocal folds	6 (12)	0 (0)	0.007
Epithelial changes	6 (12)	0 (0)	0.007
Atrophy of vocal folds	1 (2)	0 (0)	0.45
Sulcus vocalis	3 (6)	0 (0)	0.08
Cyst	1 (2)	0 (0)	0.45

Fundamental frequency in rheumatoid arthritis and control groups

Findings related to the measurement of the fundamental frequency of the two groups are provided in Table 3. As seen in Table 3, female patients with RA had statistically significant lower values in fundamental frequency than those in the control group (P<0.001). In addition, the males with RA had a higher fundamental frequency, compared to their counterparts in the control group, and this difference was statistically significant (P=0.021).

**Table 3:** Fundamental frequency in patients with rheumatoid arthritis and control group

**Variable**	**Mean (SD)**		**P-value**	**Mean (SD)**		**P-value**
Fundamental frequency	Females with rheumatoid arthritis	Females in control group	<0.001	Males with rheumatoid arthritis	Males in control group	0.021
	191.3 (30.11)	225 (16.66)		149.4 (32.43)	126.4 (14.44)	

Movement patterns of arytenoid cartilages in rheumatoid arthritis and control groups

The findings revealed symmetry in the movement of the arytenoid in 26 (52%) and 57 (90.5%) cases in the RA and control groups, respectively. In addition, 24 out of 50 participants in the RA group had overriding in arytenoid movement pattern. These values were indicative of a statistically significant difference between the two groups in terms of the patterns of movement in arytenoid (χ^2^= 21.16, df=1; P<0.001).

Patterns of glottal closure in patients with rheumatoid arthritis and control group

As seen in Table 4, the frequencies and percentages of the two groups showed a statistically significant difference regarding the complete pattern of glottal closure (P=0.00) and strained pattern of glottal closure (P=0.00). However, there was no significant difference between the RA and control groups in terms of the posterior chink (P=0.66), oval shape pattern (P=0.20), and hourglass shape pattern (P=0.32) of glottal closure.

**Table 4:** Patterns of glottal closure in patients with rheumatoid arthritis and control group

**Patterns of glottal closure**	**Patients with rheumatoid arthritis**	**Control group**	**P-value**
	**Frequency (%)**	**Frequency (%)**	
Complete	7 (14)	44 (69.8)	0.00
Posterior chink	15 (30)	15 (23.8)	0.66
Oval shape	2 (4)	0 (0)	0.20
Hourglass shape	3 (6)	1 (1.6)	0.32
Strained status	23 (46)	3 (4.8)	0.00
n	50 (100)	63 (100)	

Subglottal changes in rheumatoid arthritis and control groups

A total of 31 (62%) and 62 (98.4%) cases in the RA and control groups were found to have a normal subglot, respectively. Furthermore, 15 patients with RA had inflammation in the subglottis; however, there was no case with this sign in the control group. Stenosis was also observed in 1 and 4 (8%) cases in the control and RA groups, respectively. Based on the results of frequencies, the two groups had a statistically significant difference considering subglottal changes (χ^2^=25.98, df=2; P<0.001).

## Discussion

The current study involved the investigation of the different characteristics of vocal system, including the fundamental frequency of the voice, different structural pathologies, patterns of movement in arytenoid cartilage, patterns of glottal closure, and subglottic changes. The results revealed that the frequency of some structural changes was higher in patients with RA than in the control group. This finding is in line with those obtained by Beirith et al. One reason for this result is probably related to the disease itself and the drugs that the patients consume for this condition (11). 

There was the diagnosis of Reinke's edema in 12 (24%) patients with RA. This result is in agreement with those presented by Castro et al. also reporting that 14.8% of the patients with RA had edema in the larynx (14). There is some evidence indicating the higher prevalence of gastrointestinal disease in the RA patients than in the control group (16). The high association between gastroesophageal reflux disease and laryngeal disorders has been also reported in several studies (17-19) Regarding this, the high frequency of Reinke's edema in RA patients may be related to the higher prevalence of gastroesophageal reflux disease in this group of patients.In line with the study performed by Amernik (20), in the current research, the female patients with RA had a lower fundamental frequency than their counterparts in the control group, and this difference was statistically significant. Amernik (20) also showed that female patients with RA had a decreased fundamental frequency. This finding may be related to the higher frequency of some structural changes like Reineke's edema in this population. The decrease in fundamental frequency has been also reported in a number of studies that addressed the acoustic parameters in Reinke's edema (21, 22). Reverse findings were observed for the male patients; in this regard, the fundamental frequency was higher in the patient group than in the control group. However, there is no definite explanation for this finding. The male patients with RA only constituted 16% of the patient group with RA, and the majority of the patient group were female. Recruitment of a higher number of male patients with RA could have led to such a finding.In the present study, the patients with RA also had a higher frequency of overriding arytenoid as compared to the healthy controls. This finding is similar to the ones reported by Castro et al. Arthritis and ankylosis of cricoid joint caused by RA result in overriding arytenoid that increases with the progression of the disease (14). Additionally, a higher frequency of subglottic stenosis and inflammation was observed in the patients with RA than in the control group. There is some evidence supporting that gastroesophageal disease may be the cause of subglottic stenosis (23). Considering the high incidence of gastrointestinal disease in individuals with RA, this finding may be justified in this population. However, further evidence is required to support this finding.

There are some limitations in our research that should be considered and investigated in future studies. First, the duration of the disease was not considered as a variable. Meanwhile, the duration of the disease could have an association with the number of laryngeal manifestations in individuals with RA. Further studies are needed to study the relationship between the severity of larynx involvement and duration of RA. Second, self-report measures were not utilized in the present study. It seems that the use of subjective evaluations, such as voice activity participation profile (24) and voice handicap index (25), for investigating the effects of dysphonia on quality of life in patients with RA provides a clearer and more accurate picture of the impact of RA on the voice of the patients suffering from this disease. Third, gastroesophageal reflux disease and its relation with laryngovideostroboscopic findings in RA were not investigated in this paper. It is then suggested to conduct studies to explore the relationship between gastroesophageal disease and development of structural changes in patients with RA.

## Conclusion

As the findings indicated, the patients with RA had a higher prevalence of vocal pathologies as compared to the control group. Some patterns of glottal closure and subglottal changes were also found to be influenced by RA. In addition, the overriding arytenoid was more frequent in patients with RA than in the healthy controls. However, further studies are needed to study how these histological changes related to these conditions are made in the vocal system of RA patients.
